# Significance of Genetic Testing in Diagnosing Cholestatic Disease in Infants

**DOI:** 10.7759/cureus.103031

**Published:** 2026-02-05

**Authors:** Mahnoor Khan, Muhammad Umair Shehzad, Umaima M Khattak, Nida Zeeshan, Iqtadar Seerat

**Affiliations:** 1 Pediatric Gastroenterology and Hepatology, Pakistan Kidney and Liver Institute and Research Center, Lahore, PAK

**Keywords:** cholestatic liver disease, congenital bile acid synthesis disorder, diagnostic genetic testing, living donor liver transplant, pediatric liver diseases

## Abstract

Congenital bile acid synthesis defect type 1 (CBASD1) is an extremely rare autosomal recessive metabolic disorder caused by mutations in the HSD3B7 gene, resulting in defective bile acid synthesis and accumulation of hepatotoxic intermediates. We report a seven-month-old female infant born to consanguineous parents who presented with progressive jaundice since one month of age, severe pruritus, failure to thrive, and abdominal distension with hepatosplenomegaly. Biochemical evaluation revealed marked conjugated hyperbilirubinemia, elevated cholestatic enzymes, coagulopathy, and mild hypoalbuminemia, with low-normal gamma-glutamyl transferase levels. Elastography demonstrated advanced fibrosis (F4), and abdominal computed tomography showed cirrhotic liver changes with splenomegaly. Due to progressive liver failure despite medical therapy, the patient underwent living donor liver transplantation. The explanted liver demonstrated cholestatic hepatitis and cirrhosis, with ductular proliferation and giant-cell transformation, and no evidence of malignancy. Subsequent genetic testing identified a homozygous pathogenic mutation in the HSD3B7 gene, confirming the diagnosis of CBASD1. This case highlights the importance of early biochemical evaluation, urine bile acid profiling, and genetic testing in infants with low gamma-glutamyl transferase (GGT) cholestasis to enable timely diagnosis, continue medical optimization, and proceed with liver transplant in the future.

## Introduction

Congenital bile acid synthesis deficiencies (CBASDs) are rare autosomal recessive conditions that impair the generation of primary bile acids, causing progressive liver dysfunction and systemic complications. It comprises a heterogeneous group of rare inherited disorders caused by inborn errors of enzymes involved in the multistep conversion of cholesterol to primary bile acids, including modification of the sterol nucleus (e.g., 3β-hydroxy-Δ⁵-C₂₇-steroid oxidoreductase deficiency (HSD3B7), Δ4-3-oxosteroid 5β-reductase deficiency (AKR1D1), oxysterol 7α-hydroxylase deficiency (CYP7B1)), side-chain oxidation (e.g., CYP27A1, AMACR, ACOX2), and bile acid conjugation (e.g., BACS, BAAT) [1].

Although all subtypes share reduced or absent primary bile acids with accumulation of atypical intermediates, the clinical phenotype varies according to the affected enzyme, ranging from neonatal cholestasis and fat-soluble vitamin deficiency to progressive liver disease and, in some disorders, extrahepatic manifestations. This enzymatic and genetic heterogeneity accounts for the broad spectrum of clinical presentations observed in CBASDs and highlights the importance of accurate molecular diagnosis [1]. Congenital Bile Acid Synthesis Defect Type 1 (CBASD1) is the most common kind, caused by biallelic mutations in the HSD3B7 gene, which encodes 3β-hydroxy-Δ⁵-C27-steroid oxidoreductase. This enzyme deficiency disturbs bile acid homeostasis by inhibiting the synthesis of cholic acid (CA) and chenodeoxycholic acid (CDCA), allowing hepatotoxic C27 intermediates to accumulate [1].

Although rare, bile acid synthesis disorders can lead to persistent cholestasis in children, accounting for up to 2% of cases [1], and they are estimated to affect about one in nine million people worldwide [2]. The clinical presentation is highly diverse and nonspecific, ranging from neonatal cholestasis or coagulopathy to fat-soluble vitamin deficiencies, overlapping with other hepatobiliary pathologies. This heterogeneity often ends in delayed or missing diagnoses [2,3].

Expanding mutational data has improved understanding of CBASD1, with numerous novel HSD3B7 variants reported and marked allelic heterogeneity associated with variable clinical severity [4,5]. These observations underscore the importance of integrating biochemical and genetic approaches for accurate diagnosis. Biochemical evaluation relies on mass spectrometry-based analysis of bile acid molecular species and their precursors in body fluids, including techniques such as liquid chromatography coupled with tandem mass spectrometry (LC-MS/MS) and liquid secondary ionization mass spectrometry [1]. In settings where such analyses are not readily available, increasing use of targeted gene panels or whole-exome sequencing has enhanced the role of molecular testing, facilitating earlier diagnosis and broadening the recognized genotypic spectrum [5]. Given its rarity, nonspecific initial features, and potential progression to end-stage liver disease, reporting individual cases of CBASD1 is essential to expand the phenotypic spectrum, guide timely diagnosis, and inform management strategies.

## Case presentation

A seven-month-old girl, the only child of a consanguineous marriage, born full term (39 weeks old), was brought in with a history of jaundice since one month of age. The jaundice had worsened over time and was accompanied by intense itching for three months. She also had abdominal distension for six weeks, and frequent bloody streaked stools. Poor weight gain and concerns about her growth were additionally brought up by her parents. Upon physical examination, her vital signs were found to be stable, and she was attentive. Her weight (6.4 kg) and length (64 cm) were below the fifth percentile according to WHO growth parameters, which was consistent with failure to thrive. She had marked icterus but no dysmorphic features or skin rashes. Her abdomen was distended, with spleen palpable 3 cm below the costal margin. Neurological, respiratory, and cardiovascular examinations were unremarkable. The family history was not significant for similar manifestations and both parents were asymptomatic.

Table 1 shows typical laboratory results, such as coagulopathy, raised cholestatic enzymes, significant hyperbilirubinemia, and mild hypoalbuminemia, that are consistent with severe liver failure. The Pediatric End-Stage Liver Disease (PELD) score of 27.7 at presentation indicated severe hepatic impairment.

**Table 1:** Laboratory findings at presentation ALP: alkaline phosphatase, ALT: alanine aminotransferase, AST: aspartate aminotransferase, GGT: gamma-glutamyl transferase, INR: international normalized ratio, WBC: white blood cells. Reference ranges may vary slightly by laboratory. Laboratory investigations were performed in the institutional clinical laboratory in the Pakistan Kidney and Liver Institute and Research Center using standard automated biochemical assays according to routine clinical protocols. Age-adjusted pediatric reference ranges provided by the laboratory were used for interpretation.

Parameter	Result	Reference Range*
Total Bilirubin	15.8 mg/dL	0.3–1.2 mg/dL
Direct Bilirubin	13.2 mg/dL	<0.3 mg/dL
ALT	125 U/L	7–55 U/L
AST	320 U/L	8–48 U/L
ALP	451 U/L	45–150 U/L
GGT	31 U/L	9–48 U/L
Albumin	3.5 g/dL	3.5–5.0 g/dL
INR	2.43	0.8–1.2
WBC	21.4 ×10⁹/L	4.0–12.0 ×10⁹/L
Hemoglobin	11.1 g/dL	11–14 g/dL
Platelets	163 ×10⁹/L	150–400 ×10⁹/L

**Figure 1:**
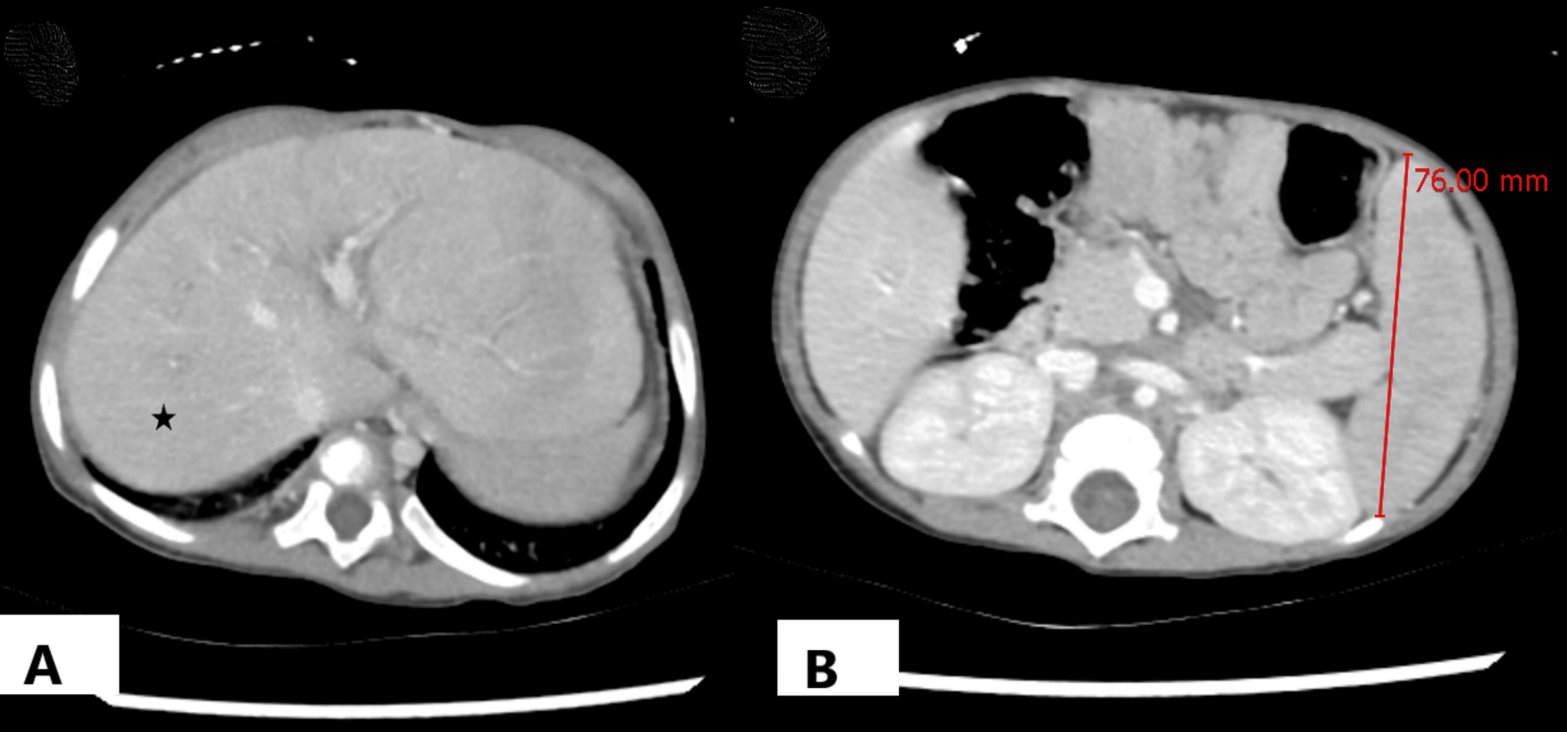
CT images of the abdomen (A) Slightly undulating hepatic margins without frank cirrhosis (asterisk shows normal liver parenchyma) associated with mild splenomegaly, (B) No focal hepatic lesion or intra-/extra- hepatic biliary dilatation was noted.

Fibro scan revealed F4 fibrosis, whereas abdominal CT revealed cirrhotic changes (Figure 1A) with splenomegaly (Figure 1B).

Liver biopsy was postponed due to coagulopathy. Progressive familial intrahepatic cholestasis (PFIC) was initially suspected based on a cholestatic profile, which included low-to-normal gamma-glutamyl transferase (GGT). She was first seen on 23 February 2023, when ursodeoxycholic acid (UDCA) was initiated at 20-30 mg/kg/day in two to three divided doses for her cholestatic jaundice. To control pruritus, cholestyramine was added at one-third of a 4g sachet, two to three times daily. She was followed up every two weeks while evaluation for a living donor transplant was undertaken, with her mother (blood group B⁺) ultimately selected as the donor.

Despite medical therapy, her liver function continued to deteriorate, and she was placed on the waiting list for a living donor liver transplant. In June 2023, she was admitted for medical optimization and transplant workup. Following a multidisciplinary team discussion, a living donor transplant was planned, and she underwent the procedure on 9 August 2023.

**Figure 2:**
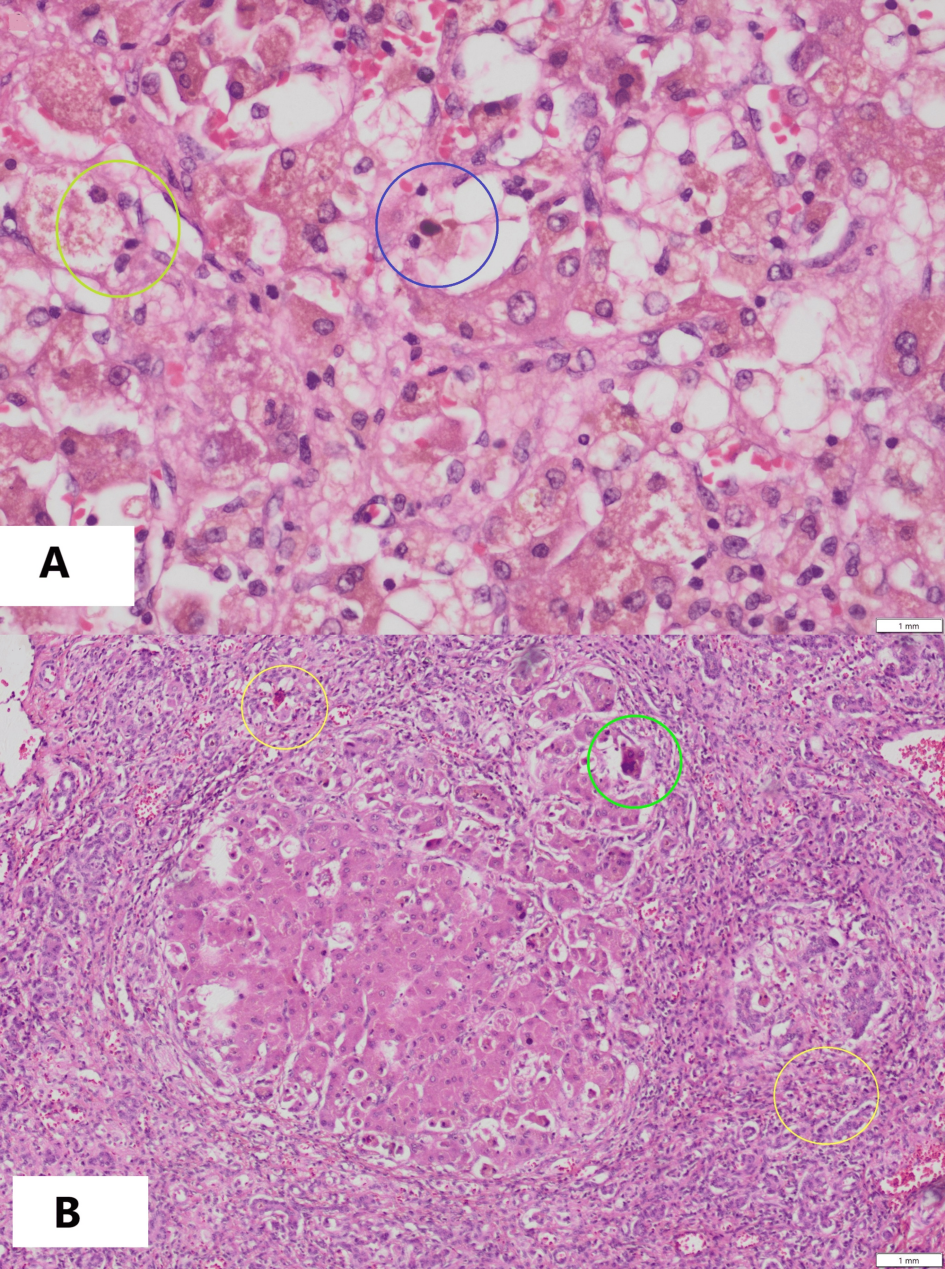
Histopathology of explanted liver (A) Feathery degeneration (yellow circle) and bile stasis/ cholestasis (blue circle), (B) Ductular proliferation (yellow circles) and multinucleated giant cells (green circle).

Postoperatively, tacrolimus-based immunosuppression was started at 0.025-0.05 mg/kg per dose twice daily, with levels checked before every fourth dose(trough level) and adjusted accordingly. Methylprednisolone was administered intraoperatively at 10 mg/kg, followed by 5 mg/kg on post-op day 1, 4 mg/kg on day 2, 2 mg/kg on day 3, 1 mg/kg on day 4, and tapered to 0.5 mg/kg thereafter. Oral prednisolone was introduced once feeding was established. Antiviral therapy was started prophylactically, as both donor and recipient were cytomegalovirus (CMV) immunoglobulin (Ig)G-positive (moderate-risk), with regular monitoring for viremia. The explanted liver demonstrated cholestatic hepatitis and cirrhosis, with ductular proliferation and giant-cell transformation (Figures 2A, 2B), and no evidence of malignancy.

**Figure 3:**
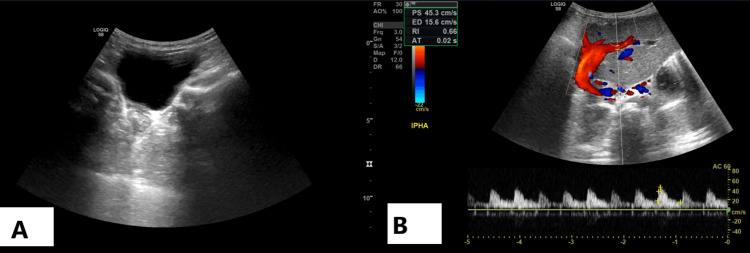
Doppler ultrasound of transplanted liver The Doppler images show normal antegrade flow in the hepatic artery, hepatic vein, and portal vein; (A) shows a normal fluid-filled urinary bladder, and (B) shows patent flow through liver vasculature.

Postoperatively, graft perfusion was confirmed by Doppler ultrasound (Figures 3A, 3B), and she was discharged two weeks after the transplant.

During follow-up, which occurred every two to four weeks for the first six months, monthly until one year, and every two to three months thereafter, she developed transient Epstein-Barr virus (EBV) reactivation and low-level CMV viremia, which were treated conservatively without sequelae. Steroids were gradually decreased over the next few months and subsequently discontinued. Tacrolimus remained within the therapeutic range, liver function tests normalized, and she was maintained on low-dose tacrolimus with regular virus surveillance. Genetic evaluation was performed on 15 April 2025 using clinical whole-exome sequencing based on next-generation sequencing technology. Analysis of the coding regions and mitochondrial genome of approximately 20,000 genes identified a homozygous likely pathogenic frameshift variant in the HSD3B7 gene located on chromosome 16 (NM_025193.3:c.963_964insC), predicted to result in a truncated protein (p.Tyr322Leufs*24), confirming congenital bile acid synthesis defect type 1 (CBASD1). She is doing well nearly two years after receiving her transplant. She weighs 13 kg, has normal liver function, stable graft performance, and no signs of rejection. She remains on low-dose tacrolimus, with routine virus surveillance and supportive care.

## Discussion

Congenital Bile Acid Synthesis Defect Type 1 (CBASD1) disrupts the conversion of 7α-hydroxycholesterol during bile acid synthesis, leading to deficient CA/CDCA output and the buildup of toxic C27 intermediates [1]. This imbalance impairs hepatocyte function and drives progressive cholestasis. Initial presentations are often misleading, mimicking viral, autoimmune, or obstructive liver disease. Prominent coagulopathy has been reported in pediatric patients with CBASD1 and may be disproportionate to the degree of cholestasis, potentially contributing to diagnostic delay [2], while atypical presentations, including fat-soluble vitamin deficiencies and bleeding manifestations, have also been described in patients with HSD3B7 deficiency [3]. Liver panels may appear deceptively normal, and pruritus can be absent, delaying recognition [2]. Our patient had severe pruritus, abdominal distension, failure to thrive, and progressive jaundice since the age of one month. She had severe cholestasis and significantly abnormal INR values, but her GGT, a biochemical indicator of CBASD1, remained low-normal. This case serves as an example of how nonspecific and sometimes paradoxical appearances can impede prompt diagnosis. With a focus on identifying mono-hydroxylated oxo-bile acids and systemic bile acid imbalance, genetic testing and mass spectrometry of urine bile acids are thought to be highly specific for CBASD1 [1]. In our instance, a homozygous HSD3B7 mutation was only genetically confirmed more than a year after liver transplantation, highlighting the difficulties and delays associated with diagnosis.

Genotypic variability further complicates the clinical picture. Previously unreported HSD3B7 mutations in children with varying severity and outcomes have been described [4], and the mutation database continues to expand, particularly in East Asian populations [5]. Such heterogeneity makes genotype-phenotype correlations unreliable and supports the need for individualized genetic counselling.

Therapeutically, cholic acid has emerged as the cornerstone of treatment. It suppresses hepatotoxic intermediates and stabilizes liver function across bile acid synthesis defects [6]. Improvements in growth and biochemical parameters have been observed after switching patients from chenodeoxycholic acid to cholic acid [7], with age-stratified dosing demonstrating greater efficacy and tolerability in pediatric cohorts [8]. Long-term outcomes are also encouraging: an open-label Phase 3 study following 53 patients over approximately four years confirmed sustained clinical and biochemical benefits, further supporting cholic acid’s role in nutritional recovery and growth promotion in children with inborn errors of bile acid metabolism [9].

Our patient progressed to decompensated liver failure despite medical therapy and required a living donor liver transplant, after which her clinical condition improved and liver function normalized. Early diagnosis and initiation of cholic acid therapy have been shown to improve biochemical markers of liver function and slow disease progression in patients with bile acid synthesis defects [7,9]. In advanced disease, progression to liver failure may occur despite medical therapy, with liver transplantation representing a life-saving option [1,10].

Patients who don't react to traditional therapy may find hope in new therapeutic approaches. Emerging therapeutic strategies targeting nuclear receptor signaling and bile acid transport pathways are currently under investigation as potential adjunctive approaches to cholic acid therapy [10]. In the future, it will be crucial to establish international registries for CBASD patients, validate supplementary treatments for cases that are resistant to cholic acid, and look into the neurocognitive effects of prolonged exposure to hazardous intermediates. Adding bile acid abnormalities to neonatal metabolic screening panels may also allow for much earlier detection and therapy, greatly improving prognosis.

Ultimately, our patient's clinical experience, from ambiguous early symptoms to delayed genetic confirmation after transplantation, demonstrates the diagnostic complexities of CBASD1. It also emphasizes the vital significance of incorporating targeted biochemical and genetic testing into pediatric hepatology practice to improve outcomes for children with this rare but curable condition.

## Conclusions

Congenital bile acid synthesis defect type 1 (CBASD1), a rare but treatable cause of infantile cholestasis, can mimic other cholestatic liver conditions, especially those characterized by low GGT levels, including progressive familial intrahepatic cholestasis. The nonspecific nature of its clinical presentation, coupled with overlapping biochemical abnormalities, frequently leads to delayed diagnosis and the progression to severe hepatic disease.

This case highlights the significance of early biochemical evaluation and genetic testing in infants with unexplained cholestasis, particularly when GGT levels are normal or low. Prompt detection of bile acid synthesis disorders allows for focused therapy with cholic acid, which can prevent liver fibrosis, reduce disease progression, and potentially eliminate the need for transplantation. Greater clinical awareness and early incorporation of genetic testing into pediatric cholestasis workups are critical for improving outcomes in this uncommon but curable illness.
